# Supplementary material for: The Systems Biology Research Tool: evolvable open-source software
Source: BMC Syst Biol. 2008 Jun 29;2:55. doi: 10.1186/1752-0509-2-55 (PMC2446383; doi:10.1186/1752-0509-2-55)
Supplement: Additional file 1 — SBRT Archive. An archive of the current version of the Systems Biology Research Tool. [file 1752-0509-2-55-S1.zip › sbrt-1.4.0/doc/developers_guide/api/sbrt/shell/text/math/package-frame.html]

sbrt.shell.text.math


sbrt.shell.text.math

Interfaces 
  
*CoeffFormat*
  
*CorrResultsFormatter*
  
*DirEdgeFormat*
  
*IndexPairFormat*
  
*IntervalFormat*
  
*LinearCombFormat*
  
*LinearCombTermFormat*
  
*MathExprFormat*
  
*MatrixElementFormat*

Classes 
  
BoundedIntervalFormatV1
  
CoeffFormatV1
  
CoeffFormatV2
  
CorrResFormatterV1
  
DirEdgeFormatV1
  
IndexPairFormatV1
  
IntervalFormatV1
  
LinCombTermFormatV1
  
LinCombTermFormatV2
  
LinearCombFormatV1
  
LinearCombFormatV2
  
MatrixElementFormatV1
  
SenseFormat
